# Investigation of Active Components of *Meconopsis integrifolia* (Maxim.) Franch in Mitigating Non-Alcoholic Fatty Liver Disease

**DOI:** 10.3390/ijms26010050

**Published:** 2024-12-24

**Authors:** Qiqin Lu, Majia La, Ziyang Wang, Jiaomei Huang, Jiahui Zhu, Dejun Zhang

**Affiliations:** 1 Research Center for High Altitude Medicine, Key Laboratory of the Ministry of High Altitude Medicine, Key Laboratory of Applied Fundamentals of High Altitude Medicine (Qinghai-Utah Joint Key Laboratory of Plateau Medicine), Laboratory for High Altitude Medicine of Qinghai Province, Qinghai University, Xining 810001, China; 2009990010@qhu.edu.cn; 2 College of Chemical Engineering, Qinghai University, Xining 810016, China; 210813000313@qhu.edu.cn (M.L.); 210813000304@qhu.edu.cn (Z.W.); 210901000123@qhu.edu.cn (J.H.); 210813000349@qhu.edu.cn (J.Z.); 3 College of Eco-Environmental Engineering, Qinghai University, Xining 810016, China

**Keywords:** *Meconopsis integrifolia* (Maxim.) Franch, non-alcoholic fatty liver disease, AMPK/SREPB-1c/PPAR-α signaling pathway, chemical constituents, molecular docking

## Abstract

Nonalcoholic fatty liver disease (NAFLD) has rapidly emerged as the most prevalent chronic liver disease globally, representing a significant and escalating public health challenge. *Meconopsis integrifolia* (Maxim.) Franch, a traditional Tibetan medicinal herb used for treating hepatitis, remains largely unexplored regarding its therapeutic potential and active components in combating NAFLD. This study first evaluated the in vitro lipid accumulation inhibitory activity of different extraction fractions of *M. integrifolia* using a HepG2 cell steatosis model. The ethyl acetate fraction was found to significantly reduce triglyceride (TG) and low-density lipoprotein (LDL) levels, inhibit lipid droplet deposition in HepG2 cells, and promote lipid metabolism balance through modulation of the AMPK/SREPB-1c/PPAR-α signaling pathway. Further analysis utilizing chromatographic techniques and nuclear magnetic resonance spectroscopy (NMR) led to the isolation of 13 compounds from the active ethyl acetate fraction. Notably, compounds **6**, **9**, **10**, **11**, **12**, and **13** were identified for the first time from this Tibetan herb. In vitro activity assays and molecular docking analyses further confirmed that the compounds Luteolin (**1**), Quercetin 3-*O*-[2‴, 6‴-*O*-diacetyl-β-d-glucopyranosyl-(1→6)-β-d-glucopyranoside] (**6**), and Quercetin 3-*O*-[2‴-*O*-acetyl-β-d-glucopyranosyl-(1→6)-β-d-glucopyranoside] (**8**) are potential key components responsible for the NAFLD-ameliorating effects of *M. integrifolia*. This study highlights the therapeutic potential of *M. integrifolia* in treating NAFLD and provides a foundation for its further development and application, underscoring its significance in the advanced utilization of traditional Tibetan medicine.

## 1. Introduction

Non-alcoholic fatty liver disease (NAFLD) is a common metabolic chronic syndrome characterized by excessive lipid accumulation in hepatocytes, leading to hepatic steatosis [1,2]. Currently, NAFLD is the most prevalent chronic liver disease worldwide [3]. Its spectrum ranges from simple steatosis to steatohepatitis with varying degrees of inflammation, fibrosis, cirrhosis, and eventually hepatocellular carcinoma [4,5]. According to recent systematic analyses, the global prevalence of NAFLD increased to 38.0% between 2016 and 2019, with a continuing upward trend [6,7]. Non-alcoholic steatohepatitis (NASH) represents a more aggressive form of NAFLD [8]. The prevalence of NAFLD rises with age, indicating that severe liver diseases are more common in elderly populations [9,10]. Consequently, global population aging is expected to further drive the burden of NAFLD, including advanced NASH and cirrhosis [11]. Additionally, the rising obesity rates among younger populations suggest that many individuals may experience NAFLD from an earlier age, prolonging disease progression [12,13]. This highlights the potential for more patients to develop advanced liver fibrosis and cirrhosis over time [14].

Moreover, NAFLD is a risk factor for the progression of various diseases, such as cardiovascular diseases, chronic kidney diseases, and malignancies, posing one of the greatest threats to human health [15,16,17,18]. The initial stage of NAFLD involves excessive hepatic lipid accumulation, emphasizing the potential therapeutic value of developing interventions that alleviate hepatic steatosis. Natural compounds in many herbal medicines have drawn significant attention for NAFLD treatment. As alternative therapies, natural products offer unique advantages in the development of effective and safe treatments for NAFLD, holding promise for therapeutic applications. Hepatic lipid accumulation results from dysregulated lipid synthesis and oxidation [19]. Excessive lipid nutrients elevate free fatty acid levels, leading to triglyceride (TG) and low-density lipoprotein (LDL) accumulation in the liver. AMP-activated protein kinase (AMPK) plays a crucial role in regulating energy metabolism [20] and is vital in lipid metabolism [21]. AMPK activation suppresses fatty acid synthesis while promoting fatty acid oxidation, positioning it as a central target for lipid-metabolism-related disorders [22]. When activated, AMPK reduces the expression of lipogenic genes such as sterol regulatory element-binding protein 1c (SREBP-1c), acetyl-CoA carboxylase (ACC), stearoyl-CoA desaturase 1 (SCD1), and fatty acid synthase (FAS). Simultaneously, it upregulates the expression of genes involved in β-oxidation, such as peroxisome proliferator-activated receptor α (PPARα) and carnitine palmitoyltransferase-1 (CPT-1) [23,24,25]. Therefore, the AMPK-mediated regulation of lipogenesis and fatty acid oxidation represents a critical strategy for improving hepatic steatosis.

The genus *Meconopsis Vig.* (Papaveraceae) comprises 49 species worldwide, predominantly distributed in the Sino-Himalayan region of East Asia, with one species native to Western Europe [26,27]. *Meconopsis* species have a long history of use in traditional Tibetan medicine, documented in Tibetan texts such as Yuewang Medical Diagnosis, Four Medical Tantras, and Jingzhu Materia Medica [28]. *M. integrifolia* (Maxim.) Franch, alongside other *Meconopsis* species such as *M. punicea*, *M. quintuplinervia*, and *M. argemonantha*, is categorized as “Obei”-type Tibetan medicine. It is characterized by its cold nature and sweet, astringent taste, with properties that clear heat, reduce dampness, and relieve inflammation and pain. Clinically, it is used to treat hepatitis, pneumonia, headaches, edema, and dysentery [27,28]. *Meconopsis* has a long history of use in traditional Tibetan medicine. The Jing Zhu Materia Medica records that *Meconopsis* flowers can “clear liver heat and lung heat” and treat throat obstructions caused by excessive heat [29]. Similarly, the *Four Medical Tantras* mention the ability of *Meconopsis* (referred to as “Oubei”) to clear liver heat [30]. The Chinese Materia Medica also documents the efficacy of “Eight-Ingredient *Meconopsis* Powder” in treating liver necrosis, hepatomegaly, and gastric bleeding [31]. Thus, *Meconopsis* is frequently included in Tibetan compound formulations for the treatment of liver diseases [30]. Notably, *Meconopsis* is a key ingredient in over 120 classical Tibetan medicinal formulations, including Ershiwu Wei Meconopsis Pill (Twenty-Five-Ingredient *Meconopsis* Pill), *Ershiwu Wei Songshi Pill* (Twenty-Five-Ingredient Pine Stone Pill), Maoban Meconopsis Bawei Formula (Eight-Ingredient *Meconopsis* Formula), Honghua Qiwei Formula (Seven-Ingredient Safflower Formula), and Zhuhuang Anle Formula (Bamboo Yellow Comfort Formula) [32]. Clinically, these formulations have been widely used for the treatment of chronic hepatitis B, viral hepatitis, and chronic severe hepatitis, demonstrating remarkable therapeutic efficacy [33,34,35,36,37,38].

Modern pharmacological studies reveal that *M. integrifolia* is rich in polyphenols and flavonoids, exhibiting anti-inflammatory, antioxidant, and hepatoprotective activities [39]. To date, compounds isolated from *M. integrifolia* primarily include flavonoids and alkaloids [40]. Among these, flavonoids such as Quercetin, apigenin, and Luteolin show significant advantages in managing metabolic syndromes, including obesity and dyslipidemia, demonstrating potential lipid-regulating effects [41,42,43]. However, the therapeutic benefits of *M. integrifolia* for NAFLD and its underlying active components remain unexplored.

To identify the active components of *M. integrifolia* with anti-NAFLD activity, this study first screened bioactive fractions with hepatoprotective effects against steatosis using in vitro cellular assays and preliminarily explored their mechanisms of action. Subsequently, the chemical constituents of the active fractions were isolated and identified. Further cellular activity evaluation and molecular docking analyses identified specific monomer compounds with NAFLD-ameliorating effects and elucidated their potential mechanisms. This study provides the first evidence of the beneficial effects of *M. integrifolia* against NAFLD and its pharmacologically active basis, offering novel insights into the advanced utilization of this traditional Tibetan medicine.

## 2. Results

### 2.1. Effects of Different Extracts of M. integrifolia on HepG2 Cell Viability

**Figure 1:**
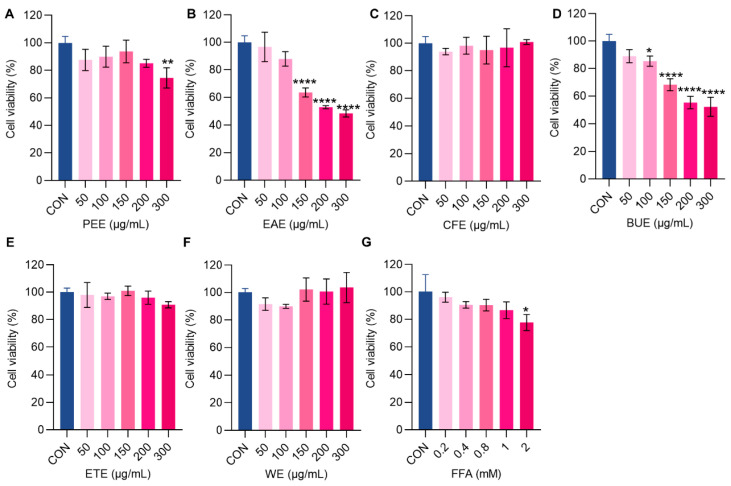
Effects of different concentrations of *Meconopsis integrifolia* extracts (**A**–**F**) and FFA (**G**) on HepG2 cell viability. PEE (petroleum ether extract), EAE (ethyl acetate extract), CFE (chloroform extract), BUE (*n*-butanol extract), ETE (ethanol extract), WE (water extract). Data are expressed as mean ± SD (*n* = 3). * *p* < 0.05, ** *p* < 0.01, and **** *p* < 0.0001 compared to the control group (CON) without drug treatments.

The effects of free fatty acid (FFA) and varying concentrations (50, 100, 150, 200, and 300 μg/mL) of different extraction fractions of *M. integrifolia* on HepG2 cell viability were evaluated using the CCK-8 assay. The results demonstrated that the petroleum ether extract (PEE) showed no significant effect on cell viability within a concentration range of 50–200 μg/mL. However, cell viability significantly decreased at concentrations exceeding 200 μg/mL (Figure 1A). The ethyl acetate extract (EAE) significantly reduced cell viability at concentrations of 150 μg/mL or higher (Figure 1B). The *n*-butanol extract (BUE) led to a significant decrease in cell viability by approximately 15% at a concentration of 100 μg/mL (*p* < 0.05, Figure 1D). The ethanol extract (ETE), water extract (WE), and chloroform extract (CFE) exhibited no significant effects on HepG2 cell viability across the tested concentration range of 50–300 μg/mL (Figure 1C,E,F). Additionally, FFA at a concentration of 2 mM significantly reduced HepG2 cell viability by approximately 22.25% (*p* < 0.05; Figure 1G). Based on these observations, an FFA concentration of 1 mM was selected to induce hepatic steatosis in subsequent experiments, and concentration gradients of 10, 25, and 50 μg/mL were chosen for the activity evaluation of different extract fractions.

### 2.2. Effects of Different Extract Fractions of M. integrifolia on FFA-Induced Lipid Accumulation in HepG2 Cells

**Figure 2:**
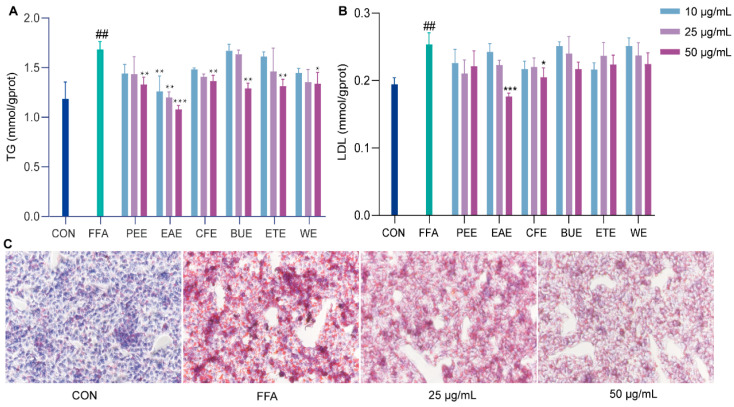
The effects of different extracts of Meconopsis integrifolia on FFA-induced lipid accumulation in HepG2 cells. (**A**,**B**) Intracellular levels of TG and LDL. (**C**) Oil Red O staining of lipid droplets (200× magnification). HepG2 cells were treated with FFA (1 mM) and/or EAE (25 and 50 μg/mL) for 24 h. PEE (petroleum ether extract), EAE (ethyl acetate extract), CFE (chloroform extract), BUE (*n*-butanol extract), ETE (ethanol extract), WE (water extract). Data are presented as mean ± SD (*n* = 3). ^##^ *p* < 0.01 compared to the CON group; * *p* < 0.05, ** *p* < 0.01, and *** *p* < 0.001 compared to the FFA group.

To evaluate the protective effects of various extracts of *M. integrifolia* against FFA-induced lipid accumulation in non-alcoholic fatty liver cells, LDL and TG levels in HepG2 cells were measured. In the FFA model group, LDL and TG levels were significantly elevated by 1.30-fold and 1.42-fold, respectively, compared to the control group, confirming the successful induction of lipid degeneration and cellular lipid accumulation. Among the extracts, only EAE reduced TG levels significantly at concentrations of 10 and 25 μg/mL by approximately 25.08% and 28.62% (*p* < 0.01). At 50 μg/mL, all extracts showed significant TG-lowering effects, and EAE and CFE also significantly reduced LDL levels by approximately 30.40% and 19.13%, respectively. Notably, EAE had the most pronounced effect in reducing LDL levels (*p* < 0.001) (Figure 2A,B). These results highlight the potent dose-dependent protective effect of EAE against lipid degeneration in hepatocytes. Oil Red O staining further demonstrated that the FFA group exhibited a significant increase in red lipid droplet areas, indicating lipid accumulation. However, treatment with 50 μg/mL of EAE significantly reduced the red lipid droplet area (Figure 2C), corroborating the lipid-lowering effect of EAE in FFA-induced cells. In conclusion, EAE was identified as the most active fraction of *M. integrifolia* for inhibiting lipid accumulation at the cellular level.

### 2.3. Protective Effects of EAE on FFA-Induced Hepatic Steatosis in HepG2 Cells

**Figure 3:**
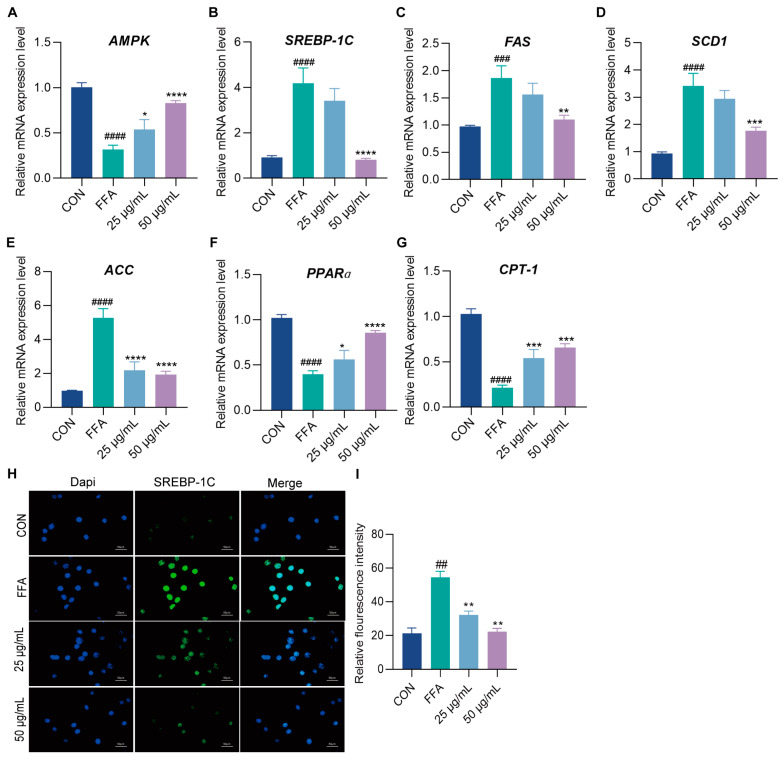
EAE regulates genes involved in lipid metabolism in HepG2 cells treated with FFA. (**A**) Relative mRNA levels of AMPK; (**B**–**E**) relative mRNA levels of SREBP1c, FAS, SCD1, and ACC (lipogenesis genes) determined by qPCR; (**F**,**G**) relative mRNA levels of PPARα and CPT1 (fatty acid oxidation genes) determined by qPCR; (**H**) immunofluorescence analysis of SREBP-1c protein expression; (**I**) quantification of SREBP-1c fluorescence intensity. Fluorescence images were captured at 400× magnification, where blue fluorescence represents the nucleus, and green fluorescence represents SREBP-1c. Data are expressed as mean ± SD (*n* = 3). ^##^ *p* < 0.01, ^###^ *p* < 0.001, and ^####^ *p* < 0.0001 compared to the CON group; * *p* < 0.05, ** *p* < 0.01, *** *p* < 0.001, and **** *p* < 0.0001 compared to the FFA group.

To further elucidate the mechanism by which *M. integrifolia* EAE inhibits TG and LDL accumulation, we analyzed the expression levels of the AMPK gene and its downstream regulatory genes in HepG2 cells. qRT-PCR analysis revealed that, compared to the control group, the expression level of the AMPK gene was significantly reduced by 68.5% in the FFA group. Treatment with 50 μg/mL of EAE markedly increased AMPK expression by 2.62-fold (Figure 3A). FFA treatment elevated the mRNA levels of SREBP-1c and its lipogenic target genes, FAS, ACC, and SCD1, by 4.55-, 1.90-, 5.40-, and 3.66-fold, respectively. However, treatment with 50 μg/mL of EAE significantly reversed the FFA-induced increase in these genes, reducing their expression levels by approximately 80.43%, 40.86%, 63.55%, and 47.99%, respectively (Figure 3B–E). In addition, we assessed the expression of genes associated with fatty acid oxidation in FFA-treated HepG2 cells. The mRNA levels of PPARα and its target gene, CPT1, were reduced by approximately 60.93% and 79.08%, respectively, upon FFA treatment. Notably, the 50 μg/mL EAE treatment restored their expression, increasing mRNA levels by approximately 2.15- and 3.06-fold, respectively (Figure 3F,G). Furthermore, immunofluorescence staining demonstrated a significant 2.57-fold increase in SREBP-1c expression in FFA-treated HepG2 cells. The EAE treatment effectively suppressed SREBP-1c expression, with reductions of approximately 40.74% and 59.22% observed at 25 μg/mL and 50 μg/mL of EAE, respectively (Figure 3H,I). These findings indicate that EAE regulates lipid metabolism and restores lipid homeostasis in FFA-induced hepatic steatosis by modulating the AMPK pathway. Specifically, EAE inhibits lipogenesis while promoting fatty acid oxidation, thereby mitigating lipid accumulation in HepG2 cells.

### 2.4. Structural Identification of Chemical Constituents Derived from EAE

**Figure 4:**
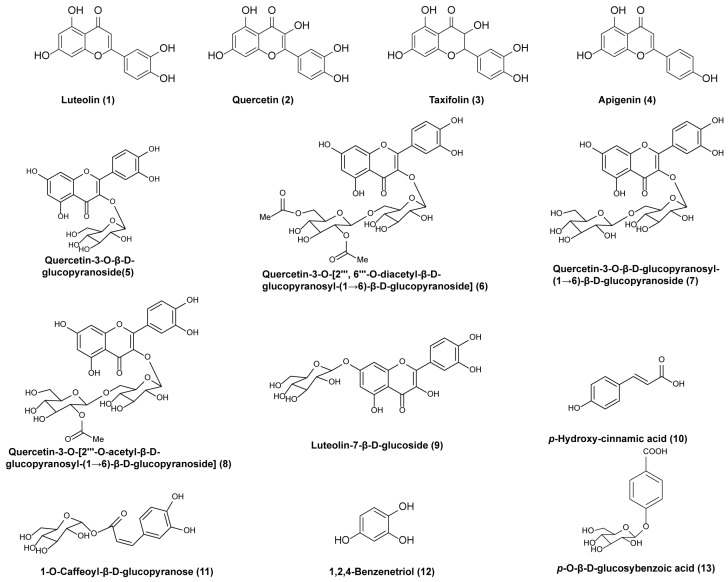
Chemical structures of compounds **1**–**13**.

Using activity-guided fractionation, the chemical constituents of the EAE were systematically isolated and identified via the NMR spectroscopic technique. Thirteen compounds were characterized (Figure 4), including eight flavonoids (compounds **1**–**9**), one phenylpropanoid (compound **10**), and three other types of compounds (compounds **11**–**13**). The identified compounds are as follows: Luteolin (**1**), Quercetin (**2**), Taxifolin (**3**), Apigenin (**4**), Quercetin-3-*O*-β-d-glucopyranoside (**5**), Quercetin-3-*O*-[2‴, 6‴-*O*-diacetyl-β-d-glucopyranosyl-(1→6)-β-d-glucopyranoside] (**6**), Quercetin-3-*O*-β-d-glucopyranosyl-(1→6)-β-d-glucopyranoside (**7**), Quercetin-3-*O*-[2‴-*O*-acetyl-β-d-glucopyranosyl-(1→6)-β-d-glucopyranoside] (**8**), Luteolin-7-β-d-glucoside (**9**), *p*-Hydroxy-cinnamic acid (**10**), 1-*O*-Caffeoyl-β-d-glucopyranose (**11**), 1,2,4-Benzenetriol (**12**), and *p*-*O*-β-d-glucosybenzoic acid (**13**). The NMR data for these compounds are provided in Supplementary Information Table S1. Notably, compounds **6**, **9**, **10**, **11**, **12**, and **13** were isolated from this Tibetan medicinal plant for the first time.

### 2.5. Effects of Isolated Compounds on In Vitro Lipid Accumulation

**Figure 5:**
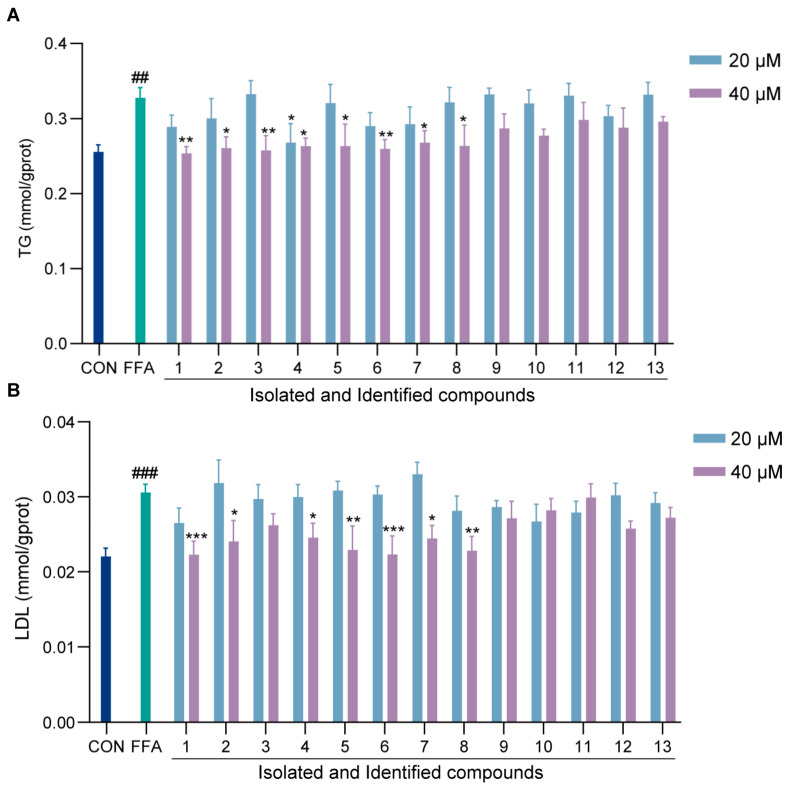
The effects of different compounds on FFA-induced lipid accumulation in HepG2 cells. (**A**) Intracellular TG levels. (**B**) Intracellular LDL levels. The compounds tested include Luteolin (**1**), Quercetin (**2**), Taxifolin (**3**), Apigenin (**4**), Quercetin-3-*O*-β-d-glucopyranoside (**5**), Quercetin-3-*O*-[2‴,6‴-*O*-diacetyl-β-d-glucopyranosyl-(1→6)-β-d-glucopyranoside] (**6**), Quercetin-3-*O*-β-d-glucopyranosyl-(1→6)-β-d-glucopyranoside (**7**), Quercetin-3-*O*-[2‴-*O*-acetyl-β-d-glucopyranosyl-(1→6)-β-d-glucopyranoside] (**8**), Luteolin-7-β-d-glucoside (**9**), *p*-Hydroxy-cinnamic acid (**10**), 1-*O*-Caffeoyl-β-d-glucopyranose (**11**), 1,2,4-Benzenetriol (**12**), and *p*-*O*-β-d-Glucosybenzoic acid (**13**). Data are presented as mean ± SD (*n* = 3). ^##^ *p* < 0.01 and ^###^ *p* < 0.001 compared to the CON group; * *p* < 0.05, ** *p* < 0.01, and *** *p*< 0.001 compared to the FFA group.

To identify the active components of EAE responsible for its therapeutic effects on NAFLD, the purified compounds were evaluated using an FFA-induced steatosis cell model. The preliminary anti-NAFLD activity was assessed by measuring the inhibitory effects of compounds **1**–**13** on TG and LDL levels in FFA-induced HepG2 cells. At a concentration of 40 μM, compounds **1**–**8** significantly reduced intracellular TG and LDL levels. Notably, Luteolin (**1**), Quercetin-3-*O*-[2‴,6‴-*O*-diacetyl-β-d-glucopyranosyl-(1→6)-β-d-glucopyranoside] (**6**), and Quercetin-3-*O*-[2‴-*O*-acetyl-β-d-glucopyranosyl-(1→6)-β-d-glucopyranoside] (**8**) exhibited particularly potent inhibitory effects (Figure 5A,B). These results highlight the potential of specific flavonoids as key bioactive compounds contributing to the lipid-lowering effects of EAE in the context of NAFLD.

### 2.6. Prediction of Anti-NAFLD Activity of Isolated Compounds Through Molecular Docking

**Figure 6:**
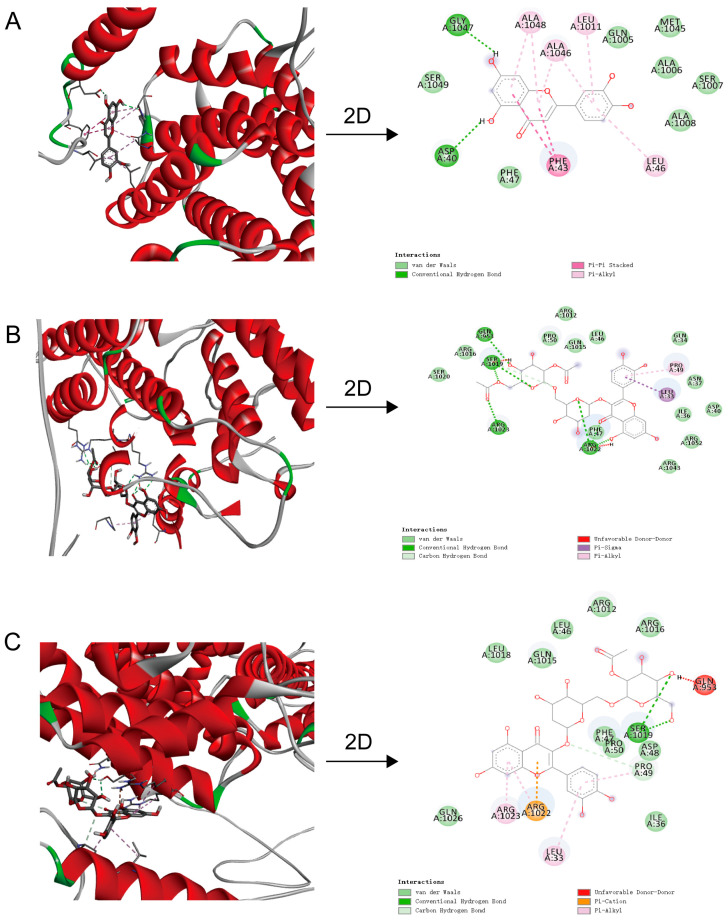
Three-dimensional and Two-dimensional visualization of the binding modes between SREBP-1c and selected compounds as determined by molecular docking. (**A**–**C**) Molecular docking of Luteolin, Quercetin-3-*O*-[2‴,6‴-*O*-diacetyl-β-d-glucopyranosyl-(1→6)-β-d-glucopyranoside], and Quercetin-3-*O*-[2‴-*O*-acetyl-β-d-glucopyranosyl-(1→6)-β-d-glucopyranoside] with SREBP-1c, respectively. Each panel presents the 3D binding conformation of the compound within the active site of SREBP-1c alongside a 2D schematic showing specific interactions, including hydrogen bonds, π-alkyl interactions, and other non-covalent forces.

To further investigate the bioactive components of *M. integrifolia* that alleviate hepatic steatosis, molecular docking was performed for the three most active compounds from in vitro experiments (**1**, **6**, and **8**) with the key regulatory factor of lipogenesis, SREBP-1c (Figure 6A–C). The docking analysis explored the potential binding modes and binding energies of the ligand molecules with the protein target, with lower binding energies indicating more stable conformations and higher receptor affinity. The results revealed strong binding affinities for all three compounds, with binding free energies below −7.0 kcal/mol, indicating robust interactions: compound **1**, binding free energy of −7.783 kcal/mol, primarily involving hydrogen bonding, π-alkyl interactions, van der Waals forces, and π-π stacking interactions; compound **6**, binding free energy of −7.433 kcal/mol, mainly driven by π-alkyl interactions, π-σ interactions, and hydrogen bonding; compound **8**, binding free energy of −8.546 kcal/mol, with interactions dominated by π-alkyl interactions, π-cation interactions, and hydrogen bonding. These molecular docking results align well with the in vitro activity screening, confirming the potential of compounds **1**, **6**, and **8** as effective inhibitors of SREBP-1c, contributing to their potential roles in regulating lipogenesis.

## 3. Discussion

Non-alcoholic fatty liver disease (NAFLD) is the most prevalent chronic liver disease worldwide, yet no specific therapeutic agents are currently available [44]. Developing drugs that reduce lipid accumulation could offer a promising approach to treating NAFLD. *M. integrifolia*, a distinctive ethnomedicine traditionally used in Tibetan regions of the Qinghai–Tibet Plateau, is known for its “liver and lung heat-clearing” properties [26,27,28,29].At present, research on this Tibetan medicine mainly focuses on population genetics, species evolution, and other aspects, while the pharmacological material basis for its anti NAFLD effect is still unclear. 

Studies have shown that a 70% ethanol extract and ethyl acetate fraction (EAE) of *M. integrifolia* exhibit protective effects against acute liver injury in mice [45] and possess strong antioxidant activity [30]. Moreover, total flavonoid extracts from *M. quintuplinervia* have demonstrated hepatoprotective effects, whereas alkaloids have shown limited efficacy [46,47], highlighting the crucial role of flavonoids in these therapeutic benefits. In this study, the EAE of *M. integrifolia* significantly reduced TG and LDL accumulation in FFA-induced HepG2 cells, demonstrating a protective effect against hepatic steatosis. Thirteen compounds were isolated from EAE, including eight flavonoids, one phenylpropanoid, and three others, with six compounds identified for the first time from this plant. Among these, compounds **1**–**8** significantly reduced lipid accumulation in vitro. This supports the hypothesis that the anti-NAFLD effects of *M. integrifolia* are closely associated with its flavonoid content. Existing studies indicate that flavonoids such as Luteolin, Quercetin, Taxifolin, and Apigenin exhibit multiple activities, including antioxidant, anti-inflammatory, and anti-tumor effects, as well as hepatoprotective and lipid-lowering properties [41,42,43,48,49,50,51,52,53]. These findings, together with the current study, underscore the pivotal role of flavonoids in *M. integrifolia*’s anti-NAFLD activity. Previous studies have demonstrated that Luteolin effectively ameliorates non-alcoholic fatty liver disease (NAFLD) through multiple pathways. Luteolin prevents NAFLD by enhancing mitochondrial function, increasing succinate dehydrogenase activity, and upregulating the AMPK/PGC-1α pathway to promote mitochondrial biogenesis. It also exerts anti-inflammatory effects by modulating the gut microbiota and reducing intestinal permeability, thereby inhibiting the progression from simple steatosis to NASH. Furthermore, Luteolin alleviates NAFLD by reducing oxidative stress, activating the PI3K/AKT signaling pathway, and improving insulin sensitivity in hepatocytes. Additionally, Luteolin-7-*O*-glucoside has been shown to activate STAT3-mediated liver cell regeneration, mitigating palmitic acid-induced hepatic injury associated with NAFLD [43,54,55].

Lipid metabolic imbalance leads to the accumulation of fat within hepatocytes, thereby promoting the development of steatosis in NAFLD [56,57]. SREBP-1c, a member of the nuclear transcription factor family, plays a central role in lipid homeostasis by indirectly regulating lipid-synthesis-related enzymes through lipid-generating target genes [19,58,59]. However, no studies to date have explored the mechanisms by which Luteolin affects lipid synthesis in NAFLD. In this study, we found that Luteolin significantly reduced TG and LDL levels in hepatocytes, alleviating lipid accumulation. Molecular docking experiments further revealed that Luteolin exhibits strong binding affinity with SREBP-1c through hydrogen bonding, π-alkyl interactions, and π-π stacking forces. These findings suggest that Luteolin may alleviate NAFLD by suppressing lipid synthesis. In future studies, we aim to further investigate the mechanisms by which Luteolin improves lipid metabolism, providing deeper insights into its therapeutic potential for NAFLD.

AMPK, a member of the serine/threonine kinase family, is a key regulator of lipid synthesis and metabolism [60]. The AMPK signaling pathway is a potential therapeutic target for NAFLD, as it modulates lipid metabolism by regulating the ACC/SREBP-1c/PPARα pathway [61,62]. AMPK activation inhibits lipogenesis while promoting fatty acid oxidation, reducing TG synthesis, and maintaining lipid homeostasis [48]. Specifically, AMPK phosphorylates and inactivates ACC, lowering malonyl-CoA levels, which reduces fatty acid synthesis and alleviates steatosis [9]. AMPK also suppresses SREBP-1c transcriptional activity, reducing lipid-synthesis-related gene expression while upregulating PPARα to stimulate fatty acid oxidation [63]. Our findings demonstrated that EAE restored AMPK activity in FFA-induced HepG2 cells, downregulating lipogenesis genes (SREBP-1c, FAS, ACC, and SCD1) while upregulating PPARα and its target gene CPT-1. These results suggest that EAE alleviates hepatic steatosis by modulating the AMPK/ACC/SREBP-1c/PPARα signaling pathway.

To identify bioactive constituents, 13 compounds were isolated from EAE using bioactivity-guided fractionation. Flavonoids, particularly Luteolin and Quercetin glycosides, exhibited potent lipid-lowering effects. Molecular docking technology predicts the binding modes between target proteins and small molecules by simulating their interactions, enabling the rapid screening of small-molecule ligands with high affinities for target proteins [64]. This technique has been widely applied in elucidating the mechanisms of action and identifying the bioactive compounds of traditional Chinese medicine in the treatment of various diseases [65,66,67,68]. SREBP-1c is a key regulator of hepatic lipogenesis, and its inactivation reduces TG synthesis and lipid accumulation [69,70]. Molecular docking further confirmed the strong binding of Luteolin and Quercetin glycosides to SREBP-1c, consistent with their in vitro activity. These results suggest that EAE’s anti-NAFLD effects may involve synergistic actions among multiple flavonoid compounds. Previous studies have highlighted diverse mechanisms underlying the anti-NAFLD effects of flavonoids: Luteolin improves NAFLD by modulating the AMPK/PGC-1α pathway, restoring gut microbiota, and inhibiting inflammatory pathways such as TLR4/NF-κB [43,54,55]. Quercetin reduces lipid accumulation by inducing autophagy, suppressing inflammatory mediators (Chemerin, CMKLR1, and NF-κB), and inhibiting inflammasome activation [71,72,73,74]. Taxifolin regulates lipid metabolism by modulating insulin signaling, SREBP-1c expression, and PPARγ activation [75,76,77]. Apigenin alleviates hepatic lipid accumulation by activating autophagy–mitochondrial pathways and suppressing NLRP3 inflammasomes [78,79,80]. These findings support the critical role of flavonoids in EAE’s therapeutic potential against NAFLD. However, further studies are needed to elucidate the mechanisms of novel compounds, such as Quercetin-3-*O*-[2‴,6‴-*O*-diacetyl-β-d-glucopyranosyl-(1→6)-β-d-glucopyranoside] and Quercetin-3-*O*-[2‴-*O*-acetyl-β-d-glucopyranosyl-(1→6)-β-d-glucopyranoside].

## 4. Materials and Methods

### 4.1. Preparation of Samples

The samples used in this study were collected from Huangnan Prefecture, Qinghai Province, China, in 2021 and identified by Prof. Zhang Dejun at Qinghai University as *Meconopsis integrifolia* (Maxim.) Franch. A voucher specimen (No. ZHANG2021-016) is deposited in the Natural Products Research Laboratory, College of Eco-Environmental Engineering, Qinghai University. Fresh *M. integrifolia* whole plants (7 kg) were air-dried and pulverized before extraction with 85% ethanol using ultrasound-assisted reflux (1:10 material-to-liquid ratio) for three cycles. The filtrates were combined and concentrated under reduced pressure to obtain an ethanol extract (ETE, 1.2 kg). The ethanol extract was suspended in water, adjusted to pH 2–3 with hydrochloric acid, and sequentially extracted three times with equal volumes of petroleum ether and ethyl acetate. The extracts were concentrated under reduced pressure to yield petroleum ether extract (PEE, 196.4 g) and ethyl acetate extract (EAE, 67.3 g). The remaining aqueous layer was adjusted to pH 9–10 with ammonia, extracted three times with chloroform, and concentrated to obtain the chloroform extract (CFE, 20.5 g). Subsequently, the aqueous layer was adjusted to pH 5–6 with hydrochloric acid, extracted three times with *n*-butanol, and concentrated to yield the *n*-butanol extract (BUE, 97.3 g). The residual aqueous solution was concentrated and dried under reduced pressure to obtain the water extract (WE, 22.6 g).

### 4.2. Cell Culture and Drug Treatment

HepG2 cells (Batch No.: CL-0103; Wuhan Procell Life Science & Technology Co., Ltd., Wuhan, China) were cultured in DMEM supplemented with 10% fetal bovine serum (FBS) and 1% penicillin–streptomycin (Batch No.: CM-0103, Wuhan Procell Life Science & Technology Co., Ltd., Wuhan, China) at 37 °C in a 5% CO_2_ incubator. When HepG2 cell confluence reached 75–85%, cells were treated with a 1 mM free fatty acid (FFA) mixture (palmitic acid–oleic acid, 1:2 ratio; palmitic acid, 10 mmol/L, Batch No.: SYSJ-KJ003; oleic acid, 12 mmol/L, Batch No.: SYSJ-KJ005; Kunchuang Technology Development Co., Ltd., Xi’an, China) for 24 h to induce hepatic steatosis [81].

Each extract fraction was dissolved in DMSO to prepare a 1 mg/mL stock solution, which was further diluted in complete culture medium to obtain working concentrations of 50, 100, 150, 200, and 300 μg/mL. Model cells were treated with the extracts at these concentrations for 24 h, and subsequent experiments were conducted. Cells cultured in DMEM without FFA served as the normal control group (CON), while those treated with FFA were the model group (FFA). Each treatment was performed in triplicate.

### 4.3. Cell Viability

HepG2 cells in the logarithmic growth phase were seeded at 1 × 10^4^ cells per well in 96-well plates (100 μL per well) and incubated for 24 h at 37 °C in a 5% CO_2_ incubator. Cells were treated with FFA (0.2, 0.4, 0.8, 1, and 2 mM) or extract fractions at various concentrations (50, 100, 150, 200, and 300 μg/mL) for 24 h. Then, 10 μL of CCK-8 reagent was added to each well and incubated at 37 °C in the dark for 2 h. Absorbance was measured at 450 nm using a microplate reader (1510 Multiskan GO, Thermo Fisher Scientific, Vantaa, Finland) to calculate relative cell viability (%) compared to the control.

### 4.4. Measurement of TG and LDL Content in HepG2 Cells

The degree of lipid accumulation in HepG2 cells was assessed by measuring intracellular TG and LDL levels using respective commercial assay kits (Jiancheng Bioengineering Institute, Nanjing, China). Cell lysates were prepared by adding lysis buffer and incubating on ice for 30 min, followed by analysis according to the kit protocols.

### 4.5. Oil Red O Staining

HepG2 cells were washed twice with PBS and fixed with 10% paraformaldehyde for 30 min. Cells were stained with Oil Red O (ORO) solution (Solarbio Technology Co., Ltd., Beijing, China) for 30 min. Stained cells were observed and imaged using a microscope (TS2R-LS Nikon, Tokyo, Japan).

### 4.6. qRT-PCR Analysis

Total RNA was extracted from cells using the Trizol reagent (Invitrogen, Carlsbad, CA, USA) and reverse-transcribed to cDNA using GeneAmp PCR System 9700 (Applied Biosystems, Thermo Fisher Scientific, Vantaa, Finland). Primers for AMPK, SREBP-1c, ACC, SCD1, PPARα, and CPT1 were synthesized by Sangon Biotech (Shanghai, China) and are listed in Supplementary Table S1. The qPCR reaction system consisted of 0.5 μL of forward and reverse primers, 5 μL of 2× PCR master mix (Arraystar SYBR^®^ Green qPCR Master Mix, Rockville, MD, USA), and 2 μL of cDNA, with water added to make an 8 μL reaction volume. qPCR was conducted using SYBR^®^ Green in the QuantStudio™ 5 System (Applied Biosystems, Thermo Fisher Scientific) under the following conditions: 95 °C for 10 min; 40 cycles of 95 °C for 10 s; and 60 °C for 60 s. This was followed by a melt curve analysis. GAPDH served as the internal control. Gene expression levels were calculated using the 2^−ΔΔCt^ method [82].

### 4.7. Immunofluorescence

HepG2 cells treated with FFA for 24 h were further treated with EAE (25 μg/mL and 50 μg/mL) for 24 h. Cells were fixed with 4% paraformaldehyde, washed, and blocked. Primary antibody against SREBP-1c (1:200) was applied at 4 °C overnight, followed by secondary antibody incubation (1:200) for 1 h. Nuclei were stained with DAPI (Beyotime Institute of Biotechnology, Shanghai, China) for 5 min. After washing with PBS, cells were observed at 400× magnification under a fluorescence microscope. Mean fluorescence intensity was quantified using the Image-Pro Plus 6.0 software (Media Cybernetics Inc., Rockville, MD, USA).

### 4.8. Chemical Composition of EAE

Chemical components of EAE were isolated using a combination of MCI gel chromatography; normal-phase and reverse-phase silica gel chromatography; Sephadex LH-20 gel chromatography; and semi-preparative HPLC (LC-20AT, SHIMADZU, Kyoto, Japan). Fractions were further analyzed using ^1^H-NMR and ^13^C-NMR techniques, and compound structures were identified by comparison with published data.

### 4.9. Screening of Compounds for Anti-NAFLD Activity

The effect of isolated compounds on TG and LDL levels in FFA-treated HepG2 cells was evaluated. Cells were treated with 1 mM FFA and individual compounds (20 or 40 µM) for 24 h. TG and LDL levels were determined as described above.

### 4.10. Molecular Docking of SREBP-1c with Active Compounds

Molecular docking was performed using the 3D structure of SREBP-1c obtained from the RCSB PDB database (https://www.rcsb.org/ (accessed on 6 May 2024). Active compounds’ 3D structures were retrieved from the PubChem database. AutoDock Tools was used for protein and ligand preparation. Docking was conducted using Discovery Studio 2019 with LibDock. Binding energy values < −4.25 kcal/mol indicated potential activity, while values < −7.0 kcal/mol suggested strong binding [83].

### 4.11. Statistical Analysis

Statistical analyses were performed using the GraphPad Prism 9.5 software. Data are presented as mean ± standard deviation (SD) from at least three independent experiments. One-way ANOVA was used for comparisons, with *p* < 0.05 considered statistically significant.

## 5. Conclusions

This study demonstrated, for the first time, that the flavonoid-rich ethyl acetate extract (EAE) of *M. integrifolia* significantly alleviates FFA-induced hepatic steatosis in vitro. EAE exerts its protective effects by modulating the AMPK/ACC/SREBP-1c/PPAR-α signaling pathway, promoting fatty acid oxidation, reducing lipogenesis, and maintaining lipid metabolic balance. Thirteen compounds were isolated from EAE, with several flavonoids identified as key bioactive constituents. Among these, Quercetin glycosides exhibited strong lipid-lowering effects, confirmed through molecular docking and in vitro experiments. These findings suggest that EAE, particularly its flavonoid components, has therapeutic potential for NAFLD and provides a scientific basis for developing *M. integrifolia* as an anti-NAFLD herbal remedy.

## Data Availability

The original contributions presented in this study are included in the article/Supplementary Material. Further inquiries can be directed to the corresponding author.

## Supplementary Materials

The following supporting information can be downloaded at https://www.mdpi.com/article/10.3390/ijms26010050/s1. References [84,85,86,87,88,89,90,91,92,93,94,95] are cited in the supplementary materials.
