# Supplementary material for: Investigation of Active Components of Meconopsis integrifolia (Maxim.) Franch in Mitigating Non-Alcoholic Fatty Liver Disease
Source: Int J Mol Sci. 2024 Dec 24;26(1):50. doi: 10.3390/ijms26010050 (PMC11719989; doi:10.3390/ijms26010050)
Supplement: Supplementary file 1 [file ijms-26-00050-s001.zip › ijms-3364309-NMR information of isolated compounds 1-13.pdf]

Supplementary information: NMR information of isolated compounds 1-13

Compound **1**: yellow powder (methanol); molecular formula: $C_{15}H_{10}O_6$; ^{1}H-NMR (500 MHz, DMSO-*d6*) δ : 12.97 (1H, s, 5-OH), 7.40 (2H, d, *J* = 6.7 Hz, H-6', H-2'), 6.90 (1H, d, *J* = 8.4 Hz, H-5'), 6.65 (1H, s, H-3), 6.44 (1H, s, H-8), 6.19 (1H, s, H-6); ^{13}C NMR (126 MHz, DMSO) δ: 166.07 (C-2), 103.89 (C-3), 183.89 (C-4), 163.26 (C-5), 100.15 (C-6), 166.38 (C-7), 95.02(C-8), 159.45 (C-9), 105.34 (C-10), 123.71 (C-1′), 114.17 (C-2′), 147.08 (C-3′), 151.04 (C-4′), 116.80 (C-5′), 120.33 (C-6′) . The above data is basically consistent with literature report [1], therefore Compound 1 is identified as Luteolin.

Compound **2**: yellow powder (methanol); molecular formula:$C_{15}H_{10}O_7$; ^{1}H NMR (500 MHz, Methanol-*d*4) δ : 7.73 (1H, d, *J* = 2.1 Hz, H-2′), 7.63 (1H, dd, *J* = 8.5, 2.2 Hz, H-6′), 6.88 (1H, d, *J* = 8.5 Hz, H-5′), 6.38 (1H, d , *J* = 2.1 Hz, H-8), 6.18 (1H, d, *J* = 2.1 Hz, H-6);^{13}C NMR (126 MHz, MeOD) δ: 148.79 (C-2), 137.26 (C-3), 177.34 (C-4), 162.53 (C-5), 99.26 (C-6), 165.59 (C-7), 94.43 (C-8), 158.25 (C-9), 104.54 (C-10), 124.17 (C-1′), 116.01 (C-2′), 146.24 (C-3′), 148.00 (C-4′), 116.24 (C-5′), 121.70 (C-6′). The above data is basically consistent with literature report[2]，therefore Compound 2 is identified as Quercetin.

Compound **3**: light yellow powder (methanol); molecular formula:$C_{15}H_{12}O_7$; ^{1}H NMR (500 MHz, Methanol-*d*4) δ: 6.96 (1H, d, *J* = 2.0 Hz, H-2′), 6.83 (1H, dd, *J* = 2.0 Hz, H-6′), 6.80 (1H, d, *J* = 8.1 Hz, H-5′) , 5.92 (1H, d, *J* = 2.1 Hz, H-8), 5.88 (1H, d, *J* = 2.1 Hz, H-6), 4.90 (1H, d, *J* = 11.5 Hz, H-2), 4.50 (1H, d, *J* = 11.5 Hz, H-3);^{13}C NMR (126 MHz, MeOD) δ :85.17 (C-2), 73.71 (C-3), 198.47 (C-4), 165.36 (C-5), 97.32 (C-6), 168.76 (C-7), 96.29 (C-8), 164.55 (C-9), 101.87 (C-10), 129.89 (C-1′), 116.09 (C-2′), 146.36 (C-3′), 147.18 (C-4′), 115.89 (C-5′), 120.92 (C-6′) . The above data is basically consistent with literature report[3]，therefore Compound 3 is identified as Taxifolin.

Compound **4**: yellow powder (methanol); molecular formula:$C_{15}H_{10}O_5$; ^{1}H NMR (500 MHz, Methanol-d_4) δ: 7.86 (2H, d, *J* = 8.8 Hz, H-2′, 6′), 6.94 (2H, d, *J* = 8.8 Hz, H-3′, 5′), 6.60 (1H, s, H-3), 6.46 (1H, d, *J* = 2.2 Hz, H-8), 6.22 (1H, d, *J* = 2.1 Hz, H-6); ^{13}C NMR (126 MHz, DMSO) δ: 164.18 (C-2), 103.29 (C-3), 182.20 (C-4), 161.90 (C-5), 99.31 (C-6), 164.67 (C-7), 94.43 (C-8), 157.77 (C-9), 104.13 (C-10), 121.62 (C-1′), 128.93 (C-2′, 6′), 116.41 (C-3′, 5′), 161.63 (C-4′).The above data is basically consistent with literature report[4]，therefore Compound 4 is identified as Apigenin.

Compound **5**: yellow powder (methanol); molecular formula: $C_{21}H_{20}O_{12}$; ^{1}H NMR (500 MHz, Methanol-*d*4) δ: 7.71 (1H, d, *J* = 2.1 Hz, H-2′), 7.58 (1H, dd, *J* = 8.4, 2.2 Hz, H-6′), 6.86 (1H, d, *J* = 8.4 Hz, H-5′), 6.38 (1H, d, *J* = 2.2 Hz, H-8), 6.20 (1H, d, *J* = 2.1 Hz, H-6); ^{13}C NMR (126 MHz, MeOD) δ: 158.48 (C-2), 135.64 (C-3), 179.51 (C-4), 163.07 (C-5), 99.90 (C-6), 166.03 (C-7), 94.73 (C-8), 159.03 (C-9), 105.72 (C-10), 123.10 (C-1′), 117.58 (C-2′), 145.93 (C-3′), 149.87 (C-4′), 116.02 (C-5′), 123.22 (C-6′), 104.33 (C-1″), 75.76 (C-2″), 78.15 (C-3″), 71.24 (C-4″), 78.42 (C-5″), 62.58 (C-6″). The above data is basically consistent with literature report[5], therefore Compound 5 is identified as Isoquercitrin.

Compound **6**: yellow powder (methanol); molecular formula: $C_{31}H_{34}O_{19}$; ^{1}H NMR (500 MHz, Methanol-*d*4) δ: 8.13 (1H, d, *J* = 2.2 Hz, H-2′), 7.74 (1H, dd, *J* = 8.6, 2.2 Hz, H-6′), 6.91 (1H, d, *J* = 8.6 Hz, H-5′), 6.46 (1H, d, *J* = 2.1 Hz, H-8), 6.22 (1H, d, *J* = 2.1 Hz, H-6), 5.14 (1H, d, *J* = 7.8 Hz, H-1″), 4.42 (1H, dd, *J* = 9.6, 8.1 Hz, H-2‴), 4.26 (1H, dd, *J* = 11.9, 2.1 Hz, H-6‴), 4.22 (1H, d, *J* = 8.1 Hz, H-1‴), 4.08 (1H, dd, *J* = 12.0, 5.5 Hz, H-6‴), 3.87 (1H, dd, *J* = 9.7, 7.8 Hz, H-2″), 3.77–

3.73 (2H, m, H-6″), 3.70 (1H, dd, *J* = 12.6, 3.0 Hz, H-4″), 3.62–3.56 (2H, m, H-3″, H-5″), 3.20 (1H, t, *J* = 9.4 Hz, H-4‴), 3.04 (1H, t, *J* = 9.3 Hz, H-3‴), 2.83 (1H, s, H-5‴), 2.04 (3H, s, Ac), 1.65 (3H, s, Ac) ^{13}C NMR (126 MHz, MeOD) *δ*:156.96 (C-2), 134.56 (C-3), 177.87 (C-4), 161.65 (C-5), 98.61 (C-6), 164.88 (C-7), 93.53 (C-8), 156.71 (C-9), 104.26 (C-10), 121.42 (C-1′), 116.49 (C-2′), 144.56 (C-3′), 148.95 (C-4′), 115.13 (C-5′), 121.20 (C-6′), 104.16 (C-1″), 71.73 (C-2″), 73.33 (C-3″), 69.39 (C-4″), 76.32 (C-5″), 67.75 (C-6″), 100.54 (C-1‴), 73.81 (C-2‴), 74.09 (C-3‴), 69.99 (C-4‴), 73.38 (C-5‴), 62.90 (C-6‴), 19.11 (Ac), 170.28 (Ac), 19.33 (Ac), 171.49 (Ac). The above data is basically consistent with literature report [6], therefore Compound 6 is identified as Quercetin 3- O-[2‴,6‴-O-diacetyl-β-D-glucopyranosyl-(1→6)-β-D-glucopyranoside].

Compound **7**: white powder (methanol); molecular formula: $C_{27}H_{30}O_{17}$; ^{1}H NMR (500 MHz, Methanol-*d*4) *δ* : 7.61 (1H, s, H-2'), 7.56 (1H, dd, *J* = 17.8, 8.5 Hz, H-6'), 6.78 (1H, d, *J* = 8.4 Hz, H-5'), 6.32 (1H, s, H-8), 6.12 (1H, s, H-6), 5.15 (1H, d, *J* = 7.6 Hz, H-1''), 4.07 (1H, d, *J* = 7.7 Hz, H-1'''); ^{13}C NMR (126 MHz, MeOD) *δ* : 157.06 (C-2), 134.17 (C-3), 177.98 (C-4) 161.60 (C-5), 98.47 (C-6), 164.57 (C-7), 93.40 (C-8), 157.47 (C-9), 104.35 (C-10), 121.68 (C-1'), 116.10 (C-2'), 144.50 (C-3'), 148.42 (C-4'), 1114.66 (C-5'), 22.10 (C-6'), 103.16 (C-1''), 74.33 (C-2''), 76.59 (C-3''), 69.91 (C-4''), 76.19 (C-5''), 68.16 (C-6''), 102.56 (C-1'''), 73.67 (C-2'''), 76.37 (C-3'''), 69.91 (C-4'''), 76.47 (C-5'''), 61.10 (C-6'''). The above data is basically consistent with literature report [7], therefore Compound 7 is identified as Quercetin-3-O-β-D-glucopy-ranosyl-(1→6)-β-D-glucopyranoside.

Compound **8**: yellow powder (methanol); molecular formula:$C_{29}H_{32}O_{18}$; ^{1}H NMR (500 MHz, Methanol-*d*4) *δ* : 7.98 (1H, d, *J* = 2.2 Hz, H-2′), 7.62 (1H, dd, *J* = 8.6, 2.2 Hz, H-6′), 6.81 (1H, d, *J* = 8.6 Hz, H-5′), 6.36 (1H, d, *J* = 2.0 Hz, H-8), 6.13 (1H, d, *J* = 2.0 Hz, H-6), 5.03 (1H, d, *J* = 7.8 Hz, H-1″), 4.37 (1H, dd, *J* = 9.5, 8.1 Hz, H-2‴), 4.13 (1H, d, *J* = 8.0 Hz, H-1‴), 3.77 (1H, dd, *J* = 9.6, 7.8 Hz, H-2″), 3.67 (3H, dq, *J* = 12.0, 3.6 Hz, H-4″, H-6″, H-6‴), 3.50 (3H, ddd, *J* = 16.3, 9.3, 4.2 Hz, H-3″, H-5″, H-6‴), 3.17 (1H, t, *J* = 9.4 Hz, H-4‴), 3.01 (1H, t, *J* = 9.3 Hz, H-3‴), 2.80 (1H, ddd, *J* = 9.7, 5.1, 2.3 Hz, H-5‴), 1.60 (3H, s, Ac); ^{13}C NMR (126M Hz, MeOD) *δ*: 156.87 (C-2), 134.55 (C-3), 177.90 (C-4), 161.55 (C-5), 98.60 (C-6), 164.84 (C-7), 93.47 (C-8), 156.96 (C-9), 104.27 (C-10), 121.25 (C-1'), 116.51 (C-2'), 144.49 (C-3'), 148.83 (C-4'), 115.04 (C-5'), 121.50 (C-6'), 104.20 (C-1''), 71.77 (C-2''), 73.42 (C-3''), 69.16 (C-4''), 75.90 (C-5''), 67.81 (C-6''), 100.69 (C-1'''), 73.91 (C-2'''), 74.30 (C-3'''), 69.94 (C-4'''), 75.79 (C-5'''), 60.82 (C-6'''), 170.39 (Ac), 19.19 (Ac). The above data is basically consistent with literature report [6], therefore Compound 8 is identified as Quercetin3-O-[2'''-O-acetyl-β-D-glucopyranosyl-(1→6)-β-D-glucopyranoside].

Compound **9**: light yellow powder (methanol); molecular formula: $C_{21}H_{20}O_{11}$; ^{1}H NMR (500 MHz, DMSO-*d*6) *δ* : 7.42 – 7.45 (2H, m, H-2′, H-6′), 6.88 (1H, d, *J* = 8.2 Hz, H-5′), 6.79 (1H, s, H-3), 6.74 (1H, s, H-6), 6.44 (1H, s, H-8), 5.08 (3H, d, *J* = 7.5 Hz, H-1″), 3.70～3.81 (6H, m, Glu-H-2″～6″)。^{13}C NMR (126 MHz, DMSO-D6) *δ* : 182.28 (C-4), 165.01 (C-7), 163.35 (C-2), 161.56 (C-5), 157.38 (C-9), 151.11 (C-4'), 146.43 (C-3'), 121.26 (C-1'), 119.68 (C-6'), 116.41 (C-5'), 113.79 (C-2'), 105.61 (C-3), 103.37 (C-10), 100.32 (1-Glc), 99.95 (C-6), 95.14 (C-8), 77.61 (4-Glc), 76.84 (3-Glc), 73.56 (2-Glc), 69.99 (5-Glc), 60.85 (6-Glc). The above data is basically consistent with literature report [8], therefore Compound 9 is identified as Luteolin 7-β-D-glucoside.

Compound **10**: light yellow powder （methanol）; molecular formula: $C_9H_8O_3$; ^{1}H NMR (500 MHz, Methanol-*d*4) *δ* : 7.59 (1H，d, *J* = 15.9 Hz, H-7), 7.46 (2H，d, *J* = 8.6 Hz, H-2, 6), 6.82 (2H, d, *J* = 8.6 Hz, H-3, 5), 6.31 (1H, d, *J* = 15.9 Hz, H-8)。^{13}C NMR (126 MHz, MeOD) *δ* : 125.96 (C-1), 129.56 (C-2, 6), 115.35 (C-3, 5), 159.62 (C-4), 144.68 (C-7) 114.94 (C-8). The above data is

basically consistent with literature report[9],therefore Compound 10 is identified as p-Hydroxy-cinnamic acid.

Compound **11**: light yellow powder (methanol); molecular formula: $C_{15}H_{18}O_9$; ^{1}H NMR (500 MHz, Methanol-*d*4) δ : 7.57 (1H, d, *J* = 15.9 Hz, H-7), 6.97 (1H, d, *J* = 1.9 Hz, H-2), 6.88 (1H, dd, *J* = 8.2, 1.9 Hz, H-5), 6.70 (1H, d, *J* = 8.2 Hz, H-6), 6.22 (1H, d, *J* = 15.9 Hz, H-8), 5.49 (1H, d, *J* = 7.8 Hz, Glc-H-1), 3.78 –3.34 (5H, m, Glc-H-2～6)。^{13}C NMR (126 MHz, MeOD) δ : 126.16 (C-1), 113.83 (C-2), 146.95 (C-3), 145.47 (C-4), 115.11 (C-5), 121.83 (C-6), 148.51 (C-7), 112.96 (C-8), 166.33 (C-9), 94.36 (Glc-C-1), 72.63 (Glc-C-2), 77.40 (Glc-C-3), 69.70 (Glc-C-4), 76.62 (Glc-C-5), 60.93 (Glc-C-6). The above data is basically consistent with literature report [10], therefore Compound 11 is identified as 1-O-Caffeoyl-β-D-glucopyranose.

Compound **12**: white powder (methanol); molecular formula:$C_6H_6O_3$; ^{1}H NMR (500 MHz, DMSO-*d*6) δ : 7.33 (1H, d, *J* = 2.0 Hz, H-5), 7.27 (1H, dd, *J* = 8.2, 2.0 Hz, H-6), 6.76 (1H, d, *J* = 8.2 Hz, H-3); ^{13}C NMR (126 MHz, DMSO) δ : 137.18 (C-1), 144.69 (C-2), 114.95 (C-3), 149.63 (C-4), 116.49 (C-5), 121.61 (C-6). The above data is basically consistent with literature report [11], therefore Compound 12 is identified as1,2,4-Benzenetriol.

Compound **13**: white powder (methanol); molecular formula:$C_6H_6O_3$; $C_{13}H_{16}O_8$; ^{1}H NMR (500 MHz, DMSO-*d*6) δ : 7.79 (2H, d, *J* = 8.4 Hz), 7.00 (2H, d, *J* = 8.5 Hz), 4.89 (1H, d, *J* = 7.2 Hz), 3.43–2.99 (6H, m); ^{13}C NMR (126 MHz, DMSO) δ : 123.90 (C-1), 130.25 (C-6, C-2), 114.87 (C-3, C-5), 159.80 (C-4), 98.99 (C-1′), 72.32 (C-2′), 75.69 (C-3′), 68.73 (C-4′), 76.25 (C-5′), 59.74 (C-6′), 166.34 (-COO-). The above data is basically consistent with literature report [12], therefore Compound 13 is identified as p-O-β-D-Glucosybenzoic acid.

References

1. Yang, L.Y.W., C.Y.; Chen, J.L.; Qiu J.l.; Du, C.X.; Wei, Y.H.; Hao, X.J.; Gu, W. Chemical constituents and bioactivitie of whole plant of *Primulina eburnea* from Guizhou. *Chin. Tradit. Herb. Drugs* **2023**, *54*, 3430–3437.
2. Lu, J.L.; Wu, M.F.; Huang, M.Y. Chemical constituents from underground parts of *Fallopia dumetorum*. *Chin. Tradit. Herb. Drugs* **2023**, *54*, 473–483.
3. Askarova, O.K.; Ganiev, A.A.; Bobakulov, K.M.; Siddikov, D.R.; Botirov, E.K.; Abdulalimov, O.; Turgunov, K.K.; Tashkhodzhaev, B. Flavonoids from the Aerial Part of *Perovskia angustifolia*. *Chem. Nat. Compd.* **2023**, *59*, 941–943.
4. Yang, S.Y.; Jiang, G.H.; Sun, Q.H.; Luo, Q.; Liu, B.; Zhan, R.; Aisa, H.A.; Chen, Y.G. Compounds from the Leaves and Stems of *Machilus salicina*. *Chem. Nat. Compd.* **2023**, *59*, 765–767.
5. Linh, N.T.T.; Thuy, T.T.; Tam, N.T.; Cham, B.T.; Tam, K.T.; Sa, N.H.; Thao, D.T.; Chinh, V.T.; Anh, N.T.H. Chemical constituents of Tard. and their-glucosidase inhibition activities. *Nat. Prod. Res.* **2022**, *36*, 3229–3233.
6. Shang, X.Y.; Wang, Y.H.; Li, C.; Zhang, C.Z.; Yang, Y.C.; Shi, J.G. Acetylated flavonol diglucosides from. *Phytochemistry* **2006**, *67*, 511–515.
7. Byun, E.; Jeong, G.S.; An, R.B.; Min, T.S.; Kim, Y.C. Tribuli Fructus Constituents Protect against Tacrine-Induced Cytotoxicity in HepG2 Cells. *Arch. Pharmacal Res.* **2010**, *33*, 67–70.
8. Lu, Y.R.; Foo, L.Y. Flavonoid and phenolic glycosides from *Salvia officinalis*. *Phytochemistry* **2000**, *55*, 263–267.
9. Zhu, T.C.; Zhu, Y.; Li, M.S.; Zhou, M.M.; Luo, J.Z.; Song, X.X.; Li, J.J.; Ouyang, Z.W.; Wang, F.F.; Qin, F. Chemical Constituents of *Selaginella moellendorffii*. *Chem. Nat. Compd.* **2022**, *58*, 122–124.
10. Li, F.; Yan, T.T.; Fu, Y.Y.; Zhang, N.L.; Wang, L.; Zhang, Y.B.; Du, J.; Liu, J.F. New Phenylpropanoid Glycosides from and Their Radical Scavenging Activities. *Chem. Biodivers.* **2021**, *18*.
11. Long, G.Q.; Wang, D.d.; Hu, G.S. Chemical constituents of Sophora flavescens and its antitumor activities in vitro. *Chin. Tradit. Herb. Drugs* **2022**, *53*, 978–984.

12. Tabata, M.; Umetani, Y.; Ooya, M.; Tanaka, S. Glucosylation of Phenolic-Compounds by Plant-Cell Cultures. *Phytochemistry* **1988**, *27*, 809–813.
