# Supplementary material for: Investigation of Active Components of Meconopsis integrifolia (Maxim.) Franch in Mitigating Non-Alcoholic Fatty Liver Disease
Source: Int J Mol Sci. 2024 Dec 24;26(1):50. doi: 10.3390/ijms26010050 (PMC11719989; doi:10.3390/ijms26010050)
Supplement: Supplementary file 1 [file ijms-26-00050-s001.zip › ijms-3364309-Table S1 Sequence of the primers used for qRT-PCR in this study.pdf]

Table S1. Sequence of the primers used for qRT-PCR in this study.

Gene		Primer Sequence (5′to 3′)
AMPK	Forward	CGGAGCCTTGATGTGGTAGG
	Reverse	GATGGTGTACTGATGACCTGG
ACC	Forward	TGAGGAACACTGAACTGGGG
	Reverse	TGGGGAATCGCTTGTCACTC
SCD1	Forward	CGCTGGCACATCAACTTCAC
	Reverse	AGGAACTCAGAAGCCCAAAGC
FAS	Forward	CTCAACAACCATGCTGGGCA
	Reverse	GGGCTTATGGCAGAATTGGC
SREBP-1c	Forward	GGACGAGCTACCCTTCGGT
	Reverse	CTGTCTCACCCCCAGCATAG
PPARa	Forward	AGCCTCAGCCAAGTTGAAGT
	Reverse	CCGAACTTGACCAGCCACAA
CPT1	Forward	GAATCGCCACGTTGGACCTA
	Reverse	TGCATGCATTCCATCGCAAG
